# Determination of Mineral, Trace Element, and Pesticide Levels in Honey Samples Originating from Different Regions of Malaysia Compared to Manuka Honey

**DOI:** 10.1155/2014/359890

**Published:** 2014-06-01

**Authors:** Mohammed Moniruzzaman, Muhammed Alamgir Zaman Chowdhury, Mohammad Abdur Rahman, Siti Amrah Sulaiman, Siew Hua Gan

**Affiliations:** ^1^Department of Pharmacology, School of Medical Sciences, Universiti Sains Malaysia, 16150 Kubang Kerian, Kelantan, Malaysia; ^2^Agrochemicals and Environmental Research Division, Institute of Food & Radiation Biology, Atomic Energy Research Establishment, Savar, Dhaka 1349, Bangladesh; ^3^Human Genome Centre, School of Medical Sciences, Universiti Sains Malaysia, 16150 Kubang Kerian, Kelantan, Malaysia

## Abstract

The present study was undertaken to determine the content of six minerals, five trace elements, and ten pesticide residues in honeys originating from different regions of Malaysia. Calcium (Ca), magnesium (Mg), iron (Fe), and zinc (Zn) were analyzed by flame atomic absorption spectrometry (FAAS), while sodium (Na) and potassium (K) were analyzed by flame emission spectrometry (FAES). Trace elements such as arsenic (As), lead (Pb), cadmium (Cd), copper (Cu), and cobalt (Co) were analyzed by graphite furnace atomic absorption spectrometry (GFAAS) following the microwave digestion of honey. High mineral contents were observed in the investigated honeys with K, Na, Ca, and Fe being the most abundant elements (mean concentrations of 1349.34, 236.80, 183.67, and 162.31 mg/kg, resp.). The concentrations of the trace elements were within the recommended limits, indicating that the honeys were of good quality. Principal component analysis reveals good discrimination between the different honey samples. The pesticide analysis for the presence of organophosphorus and carbamates was performed by high performance liquid chromatography (HPLC). No pesticide residues were detected in any of the investigated honey samples, indicating that the honeys were pure. Our study reveals that Malaysian honeys are rich sources of minerals with trace elements present within permissible limits and that they are free from pesticide contamination.

## 1. Introduction

Honey is well known for its valuable nutritional and medicinal qualities. These properties, among other benefits, are determined by the mineral components while the content of honey depends on its type and origin. The sources of the mineral components of honey are primarily its raw materials (nectar and honeydew) and the pollen grains but not the bee, as previously suggested [1].

The mineral content in honey is dependent on the natural absorption of minerals by plants from the soil and the environment [2, 3]. The absorption of minerals can also occur artificially, influenced by the composition of artificial sources such as sugar or syrup fed on by the bees. Usually, the mineral content of honey is between 0.04 and 0.20%, and it contributes to the color of the honey, which may vary from light to dark [3]. To date, approximately twenty-seven different mineral elements have been reported to be present in honey originating from nine different countries [3]. In most studies, specific groups of minerals have been reported to be present in the honey of different floral and geographical origins [1, 4–7], indicating that certain types of honey can be unique to certain countries.

Honey has been recognized as a biological indicator of environmental pollution [8]. Because honey bees readily fly within a 4 km radius of their apiaries, they access an area of approximately 50 km^2^ and can come in contact not only with the air but also with the soil and water; therefore, the concentrations of trace elements in honey can reflect the amount present in the entire region accessed [9]. The determination of trace elements in honey is therefore of high interest mainly for quality control and nutritional aspects because high levels can also be dangerous and cause toxicity.

Because of their ability to kill a wide variety of pests, pesticides play an important role in agriculture and can increase agricultural yields. However, small amounts of pesticide residues may still remain in the food chain and pose a potential risk for human health due to their ability to cause subacute and chronic toxicities. Currently, the most widely used pesticides are the organophosphorus and carbamate pesticides, which have been reported to have almost completely replaced the organochlorine pesticides [10] that are very persistent in the environment. The extensive distribution of the two groups of pesticides may, however, cause the bees feeding on the contaminated blossoms to transfer pesticide residues into honey, which may find its way to consumers [10].

Although different types of honey have been widely consumed by the general public, there is a lack of data on the mineral levels and trace element contents of Malaysian honeys. The mineral content of honey is a positive nutritional feature and, if extensively analyzed, can be used to promote a more widespread use of honey with special emphasis on its medicinal properties. In this study, we report the major minerals, the trace elements, and the pesticide content in Malaysian honeys and compare the data with honeys from other countries. To our knowledge, this is the first study to extensively report on the minerals and the trace elements and to investigate the presence of pesticide in several types of Malaysian honey samples.

## 2. Materials and Methods

### 2.1. Chemicals and Reagents

All reagents were of analytical reagent grade. Double deionized water (Milli-Q, 18.2 MΩ-cm resistivity; Millipore, Bedford, MA, USA) was used for all dilutions. Nitric acid (HNO_3_) (65%) and hydrogen peroxide (H_2_O_2_) (30%) were of Suprapur quality (Merck, Darmstadt, Germany). All glassware and plastics were cleaned by prior overnight soaking in diluted HNO_3_ (10%) before a final rinse with distilled water.

The pesticide reference standards were purchased from Dr. Ehrenstorfer GmbH (Augsburg, Germany) and were supplied in sealed vials. The certified purities of these standards ranged from 95% to 99%. Analytical grade anhydrous sodium hydrogen carbonate and anhydrous sodium sulfate (Na_2_SO_4_) were purchased from Merck (Darmstadt, Germany). Florisil (magnesium silicate, 60–100 mesh) was purchased from Sigma, St. Louis, MO, USA. The solvents acetone (BDH, UK), ethyl acetate (BDH, UK), diethyl ether (BDH, UK), n-hexane (Merck, Germany), and toluene (Merck, Germany) were of laboratory reagent grade while acetonitrile (Scharlau, EU) was of high performance liquid chromatography (HPLC) grade.

### 2.2. Honey Sample Collection

**Figure 1:**
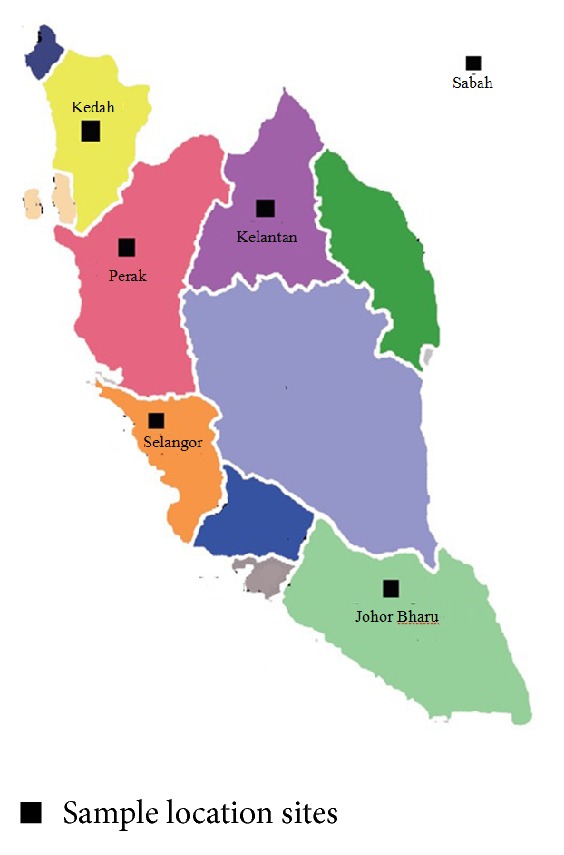
Sample collection site.

Fourteen different honey samples collected from different regions of Malaysia (Figure 1) (acacia, pineapple, gelam, longan, borneo, tualang, rubber tree, sourwood, rainforest, bitter gourd, and trigona types 1, 2, 3, and 4) were investigated (Table 1). All honey collections were conducted between July 2010 and August 2011. Manuka honey (Active 5+, Comvita, New Zealand) was used as a standard. The samples were preserved in covered airtight plastic containers and were kept at 4-5°C in a refrigerator until analysis.

### 2.3. Electrical Conductivity (EC) and Total Dissolved Solid (TDS) Contents

The EC and TDS contents were measured by an HI 98311 conductivity meter (Hanna Instruments, Mauritius) in a 20% (w/v) solution of honey suspended in Milli-Q water as described by [11]. The EC and TDS contents of each sample were analyzed in triplicate and the means were expressed as *μ*S/cm and ppm, respectively. The EC of Milli-Q water alone was less than 10 *μ*S/cm.

### 2.4. Sample Preparation for Mineral and Trace Element Contents

The standard solutions of trace elements used for calibration were prepared by diluting stock solutions (1000 mg/L) of each element supplied from Merck (Darmstadt, Germany). The serial dilutions for each of the elements analyzed were freshly prepared on the day of analysis.

#### 2.4.1. Microwave Digestion

Microwave-assisted sample digestion was performed prior to analysis to minimize the effects of the organic matrix. Briefly, 0.5 g of the honey sample was digested with 4 mL HNO_3_ (65% v/v) and 2 mL H_2_O_2_ (30% v/v) using a Multiwave 3000 microwave closed system (Anton Paar, Germany). A blank control was digested in a similar manner. The digestion program began initially at 500 W, ramped for 1 min, and was held for 4 min. The second step began at 1000 W, ramped for 5 min, and was followed by a hold period for 5 min. The third step began at a power of 1400 W and then ramped for 5 min with a hold period of 10 min. The digested samples were diluted to a final volume of 50 mL with double deionized water for the analysis of all mineral types. However, for the analysis of Na and K, a further dilution step (up to a 10-time dilution) was performed prior to analysis.

#### 2.4.2. Instrumentation

The analyses of Ca, Mg, Zn, and Fe were conducted by flame atomic absorption spectroscopy (FAAS) using an Analyst 800 atomic absorption spectrometer (Perkin Elmer, USA) equipped with an AS 800 autosampler (Perkin Elmer, USA). The analyses of Na and K were performed using flame atomic emission spectroscopy (FAES). An air-acetylene flame was used for both machines.

The analyses of As, Cd, Cu, Co, and Pb were conducted by graphite furnace atomic absorption spectroscopy (GFAAS) using a SpectrAA 220z (Varian, Belrose, Australia) with a Zeeman corrector equipped with a GTA 100z electrothermal atomizer and a Varian PSD 100 autosampler. For the graphite furnace measurements, argon was used as the inert gas. Additionally, pyrolytic coated graphite tubes with a platform were used. Hallow cathode lamps were used as sources for each of the elements investigated. Only optimal instrumental parameters, as suggested by the instrumental procedures, were used during analysis by GFAAS.

A palladium solution (500 *μ*g/mL) was used as a chemical modifier for the analysis of trace elements. The atomic absorption signal was indicated by the peak area seen in the calibration curve. The detection limits for all cases (FAAS, FAES, and GFAAS) were determined as the concentration corresponding to three times the standard deviation of 10 blanks. All specimens were run in batches that included a sample blank, a standard calibration curve, and two spiked specimens each time. For each element, five different concentrations were analyzed to obtain the calibration curve.

#### 2.4.3. Recovery Analysis

To calculate the percentage recovery, three randomly selected honey samples (acacia, pineapple, and sourwood honeys) individually spiked with known amounts of the analytical standards of the 11 elements were used as positive controls (Table 2). The mean percentage recoveries of the analyzed minerals and trace elements were then calculated based on the following equation.

Percentage recovery = [*C*_*E*_/*C*_*M*_ × 100], where *C*_*E*_ is the experimental concentration determined from the calibration curve and *C*_*M*_ is the spiked concentration.

### 2.5. Determination of Pesticide Levels

The pesticide analysis of the investigated honey samples was performed based on the method developed by [12] with some modifications. The method is detailed below. Briefly, it involved a liquid-liquid extraction of organophosphorus and carbamate pesticides from honey followed by a centrifugation step to separate the two layers. This was followed by a cleanup step using a Florisil column before HPLC analysis.

#### 2.5.1. Sample Extraction for Pesticide Residues

The honey samples (10 g) were transferred into a beaker (250 mL) and were diluted with 30 mL of purified water and 20 mL of saturated sodium chloride solution. The sample was homogenized in a water bath at 50°C for 5 min before being subsequently transferred to a screw-cap centrifuge vial (150 mL). Following the addition of dichloromethane (40 mL), the mixture was vigorously shaken for approximately 5 min followed by centrifugation for 10 min at 5000 rpm. The organic layer was collected into a 250 mL round-bottom flask and was dried by the addition of anhydrous sodium sulfate. The aqueous layer was further reextracted (twice) with dichloromethane (40 mL) followed by the addition of n-hexane (2 mL). The combined organic layers were collected before being evaporated under vacuum to approximately 2 mL on a rotary evaporator (Buchi, Switzerland) in a temperature-controlled water bath (40°C).

The evaporated sample was cleaned up with Florisil (60–100 mesh size, Sigma, USA) column chromatography with 2% diethyl ether in n-hexane. The eluent was collected and reevaporated under vacuum on a rotary evaporator with a temperature-controlled water bath (40°C) to approximately 2 mL. Finally, the evaporated sample was completely dried under a gentle stream of nitrogen before reconstitution in 1 mL of acetonitrile and injection into the HPLC system.

#### 2.5.2. HPLC Analysis for Pesticide Residues 

*(a) HPLC Machine and Parameters*. After the aforementioned sample cleanup, the samples were quantified using an LC-10 ADvp HPLC (Shimadzu) equipped with a photodiode array (PDA) detector (Shimadzu SPD-M 10 Avp, Japan) (200–800 nm). An Alltech C18 Reverse Phase (5 *μ*m, 250 × 4.6 mm) analytical column was used and maintained at 30°C in a column oven. The mobile phase was a combination of 70% acetonitrile and 30% water. The mobile phase was filtered fresh daily using a cellulose filter (0.45 *μ*m) and ran at 1.0 mL/min through the HPLC machine.

*(b) Sample Preparation*. Prior to HPLC analysis, the samples were passed through 0.45 *μ*m nylon (Alltech Assoc) syringe filters. Approximately 20 *μ*L of the samples was manually injected each time. Seven organophosphorus pesticide residues (diazinon, chlorpyrifos, acephate, dimethoate, malathion, phenthoate, and fenitrothion) and three carbamate pesticides (carbaryl, carbofuran, and carbosulfan) were analyzed in the present investigation. The calibration curves for the analyzed organophosphorus and carbamate pesticides were prepared at five different concentrations (0, 1, 5, 10, and 20 mg/L).

The precision and recovery rates of this method were determined by three consecutive analyses of three subsamples of honey spiked with known amounts of the pesticides at two different fortification levels (0.5 and 1.0 *μ*g/g) (Table 3). A blank sample was injected for every eight sample injections throughout the entire analysis. The calculation of the percentage recovery was conducted as described in the AAS analysis.

### 2.6. Statistical Analyses

The assays were performed in triplicate and the results were expressed as the mean values with standard deviations (SDs). The significant differences represented by letters were obtained by a one-way analysis of variance (ANOVA) followed by Tukey's honestly significant difference (HSD) post hoc test (*P* < 0.05). ANOVA analysis was performed by SPSS version 18.0 (IBM corporation, New York, USA) while mean and standard deviation was calculated using Microsoft Excel 2007. Principal component analysis (PCA) was performed using the Unscrambler statistical software (version 10.3, Camo Software As, Oslo, Norway).

## 3. Results and Discussion

### 3.1. EC and TDS Contents

**Figure 2:**
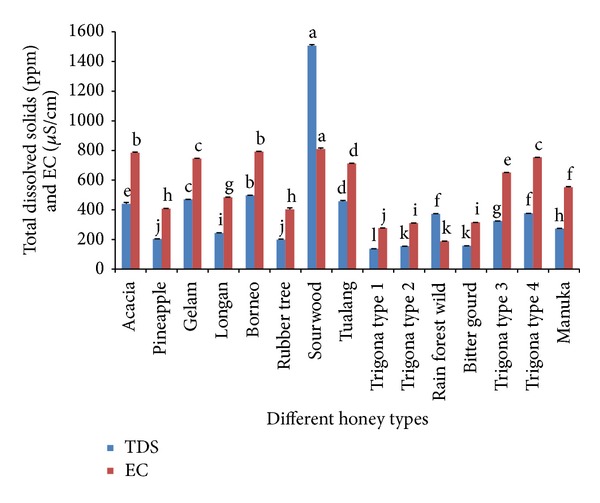
TDS and EC values of different types of Malaysian honeys. The means are compared using one-way ANOVA post hoc with multiple comparisons. In each column, the values with different letters indicate significant differences (*P* < 0.05).

EC is a key physicochemical parameter for the authentication of unifloral honeys [13]. The EC value depends on the ash and acid content in honey where the higher the content, the higher the conductivity [11]. The EC values in the investigated Malaysian honey samples ranged between 187 and 810 *μ*S/cm; thus they were generally within the recommended range (lower than 800 *μ*S/cm) except for sourwood honey, which was slightly higher (810 *μ*S/cm) (Figure 2). Overall, our results for the EC values of Malaysian honey samples are similar to those previously reported in India [14], Uruguay [15], and Morocco [16].

The TDS content is a measure of the combined content of all inorganic and organic substances in honey including the molecular, ionized, or microgranular suspended forms. The TDS content of Malaysian honeys ranged between 138.00 and 1506.33 ppm (Figure 2). Our results demonstrated that there is a correlation between the EC and the TDS content, suggesting that both parameters are good indicators for honey purity. Sourwood honey showed the highest TDS content (1506.33 ppm) as seen in the EC values as well, indicating that it is rich in both organic and inorganic substances. This could be a contribution from the organic and inorganic substances in the sourwood tree (*Oxydendrum arboreum*), which could be transferred to the nectar. However, honeydew honeys from New Zealand and Slovenia have been reported to have even higher total mineral contents at 4060 and 3680 ppm, respectively [3, 4].

### 3.2. Mineral and Trace Element Analysis

Due to the lack of an appropriate certified commercial reference material of honey, the recovery rate was measured by using known spiked concentrations of all investigated elements (As, Pb, Cu, Cd, Co, Na, K, Fe, Mg, Ca, and Zn). The consistency of the results was confirmed by measuring the recoveries of the spiked elements to sample solutions of acacia, pineapple, and sourwood honeys. The percentage recovery of the analyzed elements ranged between 91.67% and 99.07% (Table 2), indicating good accuracy, precision, and validity of the method utilized.

A total of 11 elements were analyzed. The most abundant elements were K, Na, Ca, and Fe with mean concentrations exceeding 100 mg/kg. Honey is rich in K (mean 1349.34 mg/kg), followed by Na (236.80 mg/kg), Ca (183.67 mg/kg), and Fe (162.31 mg/kg). Mg and Zn were only present at 64.46 and 43.88 mg/kg, respectively.

The mean concentration of Na in the Malaysian honey samples in the present investigation was higher than those reported for honeys from Ireland (31 mg/kg), Turkey (33 mg/kg), Spain (115 mg/kg), and Italy (96 mg/kg) [17–20]. Sourwood honey showed the highest concentration (732.16 ± 1.19 mg/kg) of Na among all honeys investigated while rubber tree honey contained the lowest amount (83.17 ± 1.17 mg/kg). Due to its high Na content, it is possible that people who are hypertensive should not consume sourwood honey in large quantities. However, this needs further investigation.

High concentrations of Na were also present in the rainforest (458.95 ± 1.34 mg/kg), bitter gourd (384.29 ± 1.82 mg/kg), tualang (268.23 ± 0.32 mg/kg), and trigona type 1 (223.18 ± 1.66 mg/kg) honeys when compared to only 190.60 ± 0.85 mg/kg present in manuka honey. The concentration of Na in the present study is also much higher than the acacia honey from Italy (12.86 mg/kg). This discrepancy may be from the geographical variation in the sources of honey [17].

The Malaysian honey samples showed a wide range of K contents (between 413.63 and 4026.40 mg/kg) (Table 4), with approximately 60% of the values exceeding 1000 mg/kg. The wide range reported for K is similar to honey samples from the Siena County region (Italy) (147 to 4136 mg/kg) [21]. Overall, the mean concentration of K in Malaysian honeys was higher than those reported from Anatolia, Turkey (296 mg/kg) [20], the Latium region, Italy (472 mg/kg), [17], Ireland (566 mg/kg) [18], the County region, Italy (1195 mg/kg) [21], and Spain (1124 mg/kg) [19], but the levels were lower than those reported for a different set of honeys from Spain (1778 mg/kg) [6].

Similar to Na content, sourwood honey also contained the highest concentration of K (4026.40 ± 3.39 mg/kg) among all honey types while rainforest honey had the lowest amount (413.63 ± 1.94 mg/kg). The high contents of Na and K in sourwood honey may contribute to the higher EC and TDS content seen earlier in sourwood honey. Other types of Malaysian honey such as trigona types 2 and 4, tualang, borneo, and gelam honeys are also rich in K (2417.43, 1971.75, 1576.40, 1459.33, and 1363.40 mg/kg, resp.) when compared to only 1229.73 mg/kg in manuka honey. One possible reason for the higher concentrations of Na and K exhibited by sourwood honey may be the presence of greater amounts of these two minerals in the leaves of sourwood tree (*Oxydendrum arboreum*) as reported by [22], which may be transferred to the nectar. The fact that sourwood honey contains both high Na and high K makes it less dangerous for the hypertensives to consume this honey type.

High concentrations (65.80 to 567.27 mg/kg) of Ca were also observed among all the investigated Malaysian honeys with the mean value (183.67 mg/kg) higher than the honeys reported for Southeast Anatolia, Turkey (51 mg/kg) [20], Italy (47.7 mg/kg) [17], Ireland (111 mg/kg) [18], and Spain (113 mg/kg) [19]. Among all investigated honey types, manuka honey showed the highest Ca concentration (809.73 mg/kg). Due to the presence of its high Ca content, it would be interesting in future studies to investigate the health benefits of long term manuka honey intake as a strategy to prevent osteoporosis.

Rainforest honey showed the second highest concentration (567.27 mg/kg) of Ca, followed by bitter gourd honey (358.27 mg/kg), gelam honey (275.77 mg/kg), and trigona honey type 2 (202.60 mg/kg). Interestingly, there are some similarities between the Ca concentrations of some types of Malaysian honeys and those coming from other parts of the world. For instance, the Ca concentration of bitter gourd honey is similar to that of honeydew honey from Italy (356 mg/kg) [21], while trigona honey type 2 has a similar Ca concentration when compared to the sulla honey (207 mg/kg) [21]. Gelam honey has a similar Ca concentration to clover honey (274 mg/kg) as reported by [21].

Fe was the fourth most abundant element in the Malaysian honeys, with contents ranging from 55.83 to 233.00 mg/kg (mean 162.31 mg/kg). Approximately 86% of the investigated honey samples, including manuka honey (216.97 mg/kg), contained an Fe content that exceeded 100 mg/kg. The highest Fe concentrations were found in rubber tree honey (233 mg/kg), while borneo (96.20 mg/kg) and sourwood (55.83 mg/kg) honeys had the lowest contents (Table 4). A possible reason for the higher Fe content in rubber tree honey may be the presence of higher Fe levels in the shoots of the rubber tree (*Hevea brasiliensis*), as reported previously by [23], which may be transferred to the nectar. Honey samples from Italy (3.07 mg/kg) [21], Spain (9.19 mg/kg) [6], Turkey (6 mg/kg) [20], Ireland (8 mg/kg) [18], and India (44.98 mg/kg) [24] reported lower Fe contents. Because rubber tree honey contained the highest Fe level when compared to other types of Malaysian honeys, it should be the honey of choice for anemics and may also be recommended for consumption by menstruating women during their menstrual periods.

The concentrations of Mg in the present study ranged from 21.83 to 199.33 mg/kg (Table 4), which is similar to that of Italian honeys (between 22.2 and 159 mg/kg; mean 56.7 mg/kg) as reported by [21]. Sourwood honey contained the highest concentration of Mg among all honeys investigated, while borneo and acacia honeys contained the lowest. A high concentration of Mg was also exhibited by trigona honey types 2 and 4 (137.93 and 164.13 mg/kg, resp.), which are similar to the honeys from Spain (136 mg/kg) [6].

The concentration of Zn in the investigated Malaysian honeys was between 4.70 and 173.77 mg/kg. Among all honeys investigated, rainforest honey is the richest in Zn (173.77 mg/kg). High concentrations of Zn were also shown by the bitter gourd and trigona types 1, 2, and 4 honeys as well as the rubber tree honey. Zn is a mineral important for wound healing and treating skin conditions such as acne or eczema and is important for the health of the prostate. Other reported levels of Zn in honeys across the world include those from Italy (3.1 mg/kg), Spain (3.9 mg/kg), Turkey (2.7 mg/kg), Ireland (5 mg/kg), and India (12.69 mg/kg) [17–21, 24]. Generally, the differences in mineral contents are attributed to the different geographical and botanical origins of the honeys [25].

As a summary, sourwood honey contained the highest Na, K, and Mg, which may contribute to its high EC and TDS values. Manuka honey is rich in Ca while rubber tree honey is rich in Fe. Rainforest honey contained high Zn levels. On the whole, Malaysian honeys are rich in minerals. Some of these elements are important components of enzymes, such as Fe, Mg, and Zn, which help in a variety of metabolic reactions in the body, while Ca and Mg can play important roles in bodily functions [26]. Consumption of such honeys can provide a good source of the essential minerals required for proper growth and bodily function. The data from our investigation suggest that certain types of honeys may be more useful for specific conditions due to differences in the presence of minerals.

It is very important to analyze the contents of trace elements in the honey samples because of their toxicity and because they can be transported through the root system into nectar or to the surfaces of the leaves. Generally, the trace element contents in Malaysian honey were low and comparable with the contents in honey from uncontaminated areas [27, 28], indicating that the investigated honeys are of good quality. The relative concentrations of other trace elements investigated in the honey samples decreased in the following order: Cu > Cd > Pb > Co > As.

In the present study, the Cu concentrations ranged from 0.00 to 2.93 mg/kg (mean 2.28 mg/kg). The highest Cu level was 2.93 mg/kg in trigona honey type 4, but Cu was not detected in trigona honey types 1 and 2. Nevertheless, the levels are still within the provisional tolerable daily intake (PTDI) for Cu (3 mg), which is the limit for metal intake based on the body weight of an average 60 kg adult [27, 29]. Generally, Cu enters food through environmental contamination, through mineralization by crops, or from the use of fungicides, bactericides, and herbicides [30].

The levels of Cu in Malaysian honey are lower than the honeys from Croatia (36.0 to 41.2 mg/kg) [27] and India (3.43 mg/kg) [24] but are higher than the honeys from Italy (647.00 *μ*g/kg and 0.81 mg/kg) [21, 31], respectively, Greece (0.14–0.48 mg/kg) [9], and the Lazio region (0.3 mg/kg) [17]. The level of Cu in Malaysian honey is similar to that in the honeys from Slovenia (1.4–2.7 mg/kg) [4], Turkey (0.23–2.41 mg/kg) [28], and Ireland (2.0 mg/kg) [18].

The level of Cd in the Malaysian honey samples ranged from 0.00 to 1.03 mg/kg, which was lower than that reported for manuka honey (1.01 mg/kg) or the honeys from Turkey (0.90–17.90 mg/kg) [28]. The levels are, however, higher than the honeys from Greece (0.08–0.22 mg/kg) [9] and India (0.25 mg/kg) [24]. Moreover, the level of Cd in manuka honey was 1.10 mg/kg.

Because of the toxicological importance of Pb, many studies have been conducted to investigate the level of this element in honeys from several countries [4, 27, 28]. Among all honey samples investigated, acacia honey had the highest Pb concentration (1.017 mg/kg). The relatively higher concentrations of Pb in acacia honey may be due to the position of the hives in the areas close to highways and railways, which is often the case [27]; however, this needs further investigation. The mean level of Pb in the Malaysian honey samples is 0.36 mg/kg, which is still within the provisional tolerable weekly intake (PTWI) of Pb for adults (3 mg) recommended by the World Health Organization. However, a lower recommended level of 0.3 mg is suggested for babies, children, and the elderly [29], making some of the honeys unsuitable to be consumed by these age groups. Furthermore, babies and children may be more sensitive to the botulinum spore content. Honey samples from other countries such as Croatia (10.0–841.0 *μ*g/kg) [27] and Italy (28.2–304.0 *μ*g/kg) [21] have also been reported to contain Pb, though they are within the recommended levels.

Low concentrations of Co (0.0–0.107 mg/kg) were observed in all investigated Malaysian honey samples, which was lower than that previously reported for Indian honey (1.22 mg/kg) [24] and honeys from Southeastern Anatolia, Turkey (1 mg/kg) [20]. Overall, our data indicate that there are low levels of Co contamination in the Malaysian honey samples.

The levels of As ranged from 0.027 to 0.126 mg/kg with a mean concentration of 0.068 mg/kg (Table 5). The As highest level (0.256 mg/kg) was found in the manuka honey while the lowest (0.027 mg/kg) was in trigona honey type 2. Because the total As content in manuka honey was high, further testing is recommended to determine the amount of organic and inorganic As. A very high As content has been reported in some honeys from Slovenia (1.24–1.49 mg/kg) [4], which may be from environmental contamination.

**Figure 3:**
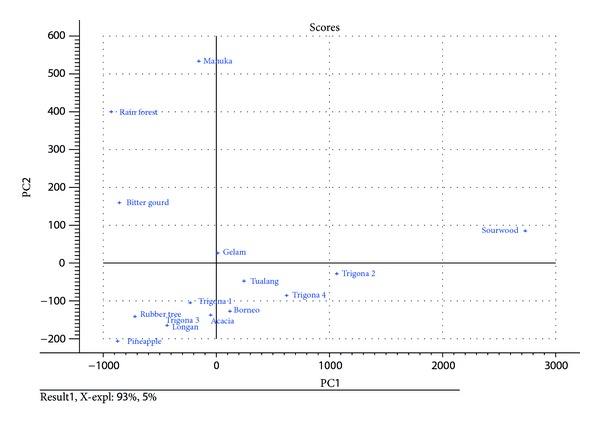
PCA analysis for different types of Malaysian honeys.

A matrix (15 × 11) with rows representing the different analyzed honey samples (objects) and columns corresponding to the content of the 11 investigated variables (minerals and trace elements) was constructed for the PCA. Chemometric analyses were performed in order to discriminate the honey samples based on their mineral contents. Based on PCA, different honey samples were found to vary not only in their geographic origins, but also in their mono- or multifloral characteristics (Table 1). PCA analysis also indicated clear separations between samples obtained from several different geographic areas in Malaysia (Figure 3). In addition, manuka honey originating from New Zealand was clearly discriminated from the rest of the honey samples from Malaysia. In this two-dimensional representation, the second component (PC1, 93% of explained variance) was mainly associated with potassium, while the explained variance of the second component (PC2, 5%) allowed good discrimination for sourwood honey. Sourwood, bitter gourd, rain forest, and gelam honeys were also well discriminated from the other types of honey indicating their marked differences in the geographical and botanical (floral) origin. However, using two principal components, some of the honey samples (borneo, tualang, and trigona types 2 and 4) occupied the same positions in the projection plot while another group of honeys (acacia, rubber tree, pineapple, longan, and trigona types 1 and 3) appeared in the same quadrant. Based on these facts, mineral contents may represent a major discriminating strength based on their floral sources.

### 3.3. Pesticide Analysis

The presence of selected pesticide residues was also investigated to determine if any contamination has occurred, especially with the flower blossoms, which may occur during the harvesting process. The most common pesticides used in the past two decades on crops and vegetables in many countries including Malaysia are organophosphorus pesticides and carbamate pesticides, as organochlorine pesticides are largely banned in many countries due to their highly persistent nature in the environment [10]. Therefore, we have selected seven organophosphorus and three carbamate pesticide residues for the investigation.

The percentage recovery of the pesticides was high (between 73% and 83% at a spiking concentration of 0.5 *μ*g/g and between 70% and 84% at 1 *μ*g/g), indicating that the method is suitable (Table 3). No pesticide residues have been detected in any of the investigated Malaysian honey samples, which indicates that the samples are pure or are collected from uncontaminated areas.

## 4. Conclusion

Our study indicated that different types of honey contain different minerals and trace elements. As a summary, sourwood honey contained the highest Na, K, and Mg, which may contribute to its high EC and TDS values. Manuka honey is rich in Ca while rubber tree honey is rich in Fe. Additionally, rainforest honey contained high Zn levels. Principal component analysis indicated clear discrimination among the different honey samples based on their mineral and trace element contents. Overall, our results indicated that the investigated Malaysian honeys are abundant sources of minerals that are essential for the human diet, growth, and health. The levels of the trace elements were within the permissible limit set by the FAO/WHO and there was no contamination of the honeys with pesticide residues. Therefore, the investigated Malaysian honeys are pure and provide a good source of minerals.

## Figures and Tables

**Table 1:** Source, floral type, and location of the analyzed Malaysian honeys.

Type of honey	Floral type (bee species)	Local tree (scientific) name	Location or supplier's name
Acacia honey	Monofloral (*Apis mellifera*)	Forest mangrove or mangium tree *(Acacia mangium) *	Koperasi Alnoor, Johor Bharu
Pineapple honey	Monofloral (*Apis mellifera*)	Pineapple (*Ananas comosus) *	Agriculture Dept.: Usahawan Lebah Madu, Putrajaya
Gelam honey	Monofloral (*Apis dorsata*)	Gelam tree (*Melaleuca cajuputi*)	The Federal Agriculture Marketing Authority (*FAMA*), Kedah
Longan honey	Monofloral (*Apis mellifera*)	Longan tree (*Dimocarpus longan*)	Beekeeper from Perak
Borneo tropical honey	Monofloral (*Apis cerana*)	Forest mangrove or mangium tree *(Acacia mangium) *	Korporasi Pembangunan Desa, Sabah
Rubber tree honey	Monofloral (*Apis mellifera*)	Rubber tree (*Hevea brasiliensis*)	Beekeeper from Perak
Sourwood honey	Monofloral (*Apis mellifera*)	Sourwood tree or Appalachian lily tree (*Oxydendrum arboreum) *	Beekeeper from Perak
Tualang honey	Multifloral (*Apis dorsata*)	Tualang tree (*Koompassia excelsa*)	The Federal Agriculture Marketing Authority (*FAMA*), Kedah
Trigona type 1 (dark in color)	Multifloral (stingless bees or *Trigona*sp.)	Local fruits	Trigona beekeepers from Lenggong, Perak
Trigona type 2 (light in color)	Multifloral (stingless bees or *Trigona *sp.)	Local fruits	Trigona beekeepers from Lenggong, Perak
Rain forest honey	Monofloral (*Apis mellifera*)	Rain forest mangium tree *(Acacia mangium) *	“The Bee shop,” Johor Bharu
Bitter gourd honey	Monofloral (*Apis mellifera*)	Bitter gourd* (Momordica charantia) *	“The Bee shop,” Johor Bharu
Trigona type 3 (light in color)	Monofloral (stingless bees or *Trigona *sp.)	Starfruit or carambola tree *(Averrhoa carambola) *	MARDI, Serdang, Selangor
Trigona type 4 (dark in color)	Monofloral (stingless bees or *Trigona *sp.)	Starfruit or carambola tree *(Averrhoa carambola) *	MARDI, Serdang, Selangor

**Table 2:** Recovery analysis for the analyzed elements in the spiked honey samples.

Type of element	Spiked value (*μ*g/kg)	*Measured value Mean ± SD (*μ*g/kg)	Recovery (%)
As	10	9.41 ± 0.03	94.13
Pb	10	9.17 ± 0.06	91.67
Cu	50	49.48 ± 0.07	98.95
Cd	10	9.89 ± 0.02	98.90
Co	20	19.71 ± 0.09	98.57
Na	50	49.25 ± 0.04	98.50
K	50	49.85 ± 0.01	99.70
Fe	50	49.09 ± 0.05	98.17
Mg	50	49.91 ± 0.01	99.81
Ca	20	19.76 ± 0.09	98.78
Zn	20	19.81 ± 0.03	99.07

*Mean value of triplicates.

**Table 3:** Recovery analysis for the analyzed pesticides in the spiked honey samples.

Compound	Amount (ng) in HPLC*		Recovery %
	Spiked	Measured	
Diazinon	100	81	81.00
	200	160	80.00
Chlorpyrifos	100	82	82.00
	200	168	84.00
Acephate	100	80	80.00
	200	166	83.00
Dimethoate	100	79	79.00
	200	152	76.00
Malathion	100	73	73.00
	200	140	70.00
Phenthoate	100	80	80.00
	200	164	82.00
Fenitrothion	100	83	83.00
	200	164	82.00
Carbaryl	100	83	83.00
	200	162	81.00
Carbofuran	100	75	75.00
	200	154	77.00
Carbosulfan	100	76	76.00
	200	158	79.00

*Mean value of triplicates.

**Table 4:** Mineral contents of analyzed Malaysian honeys.

Type of honey	Na mg/kg	K mg/kg	Ca mg/kg	Fe mg/kg	Mg mg/kg	Zn mg/kg
Acacia	173.33 ± 0.95	1292.28 ± 4.63	106.83 ± 8.83	162.43 ± 7.66	23.27 ± 1.14	12.40 ± 0.79
Pineapple	111.29 ± 1.01	473.68 ± 5.20	74.60 ± 9.56	173.60 ± 6.29	36.63 ± 0.86	11.73 ± 0.55
Gelam	196.84 ± 1.65	1363.40 ± 4.81	275.77 ± 8.98	142.37 ± 4.19	31.63 ± 1.42	29.23 ± 0.81
Longan	95.94 ± 0.08	906.35 ± 3.32	118.07 ± 11.54	164.30 ± 6.34	35.47 ± 0.68	26.47 ± 1.04
Borneo	180.23 ± 1.73	1459.33 ± 1.87	119.80 ± 10.05	96.20 ± 4.33	21.83 ± 1.25	4.70 ± 0.79
Rubber tree	83.17 ± 1.17	630.28 ± 3.22	141.17 ± 12.56	233.00 ± 10.07	59.07 ± 0.45	56.13 ± 1.00
Sourwood	732.16 ± 1.19	4026.40 ± 3.39	65.80 ± 10.17	55.83 ± 6.46	199.33 ± 2.73	27.97 ± 0.85
Tualang	268.23 ± 0.32	1576.40 ± 0.85	165.10 ± 14.16	128.13 ± 6.18	35.03 ± 0.46	13.20 ± 0.61
Trigona type 1	223.18 ± 1.66	1109.28 ± 1.80	106.00 ± 9.51	214.63 ± 10.78	26.37 ± 1.54	103.47 ± 1.82
Trigona type 2	171.68 ± 0.95	2417.43 ± 2.02	202.60 ± 12.41	141.50 ± 16.60	137.93 ± 2.03	18.63 ± 1.91
Rain forest	458.95 ± 1.34	413.63 ± 1.94	567.27 ± 13.03	210.33 ± 10.84	44.97 ± 0.76	173.77 ± 2.05
Bitter gourd	384.29 ± 1.82	472.70 ± 2.40	358.27 ± 10.60	162.37 ± 10.22	44.87 ± 0.97	56.80 ± 1.73
Trigona type 3	160.68 ± 2.37	777.95 ± 1.34	113.60 ± 8.40	165.40 ± 9.89	41.90 ± 1.06	12.30 ± 0.96
Trigona type 4	117.23 ± 1.73	1971.75 ± 1.06	156.47 ± 10.86	222.20 ± 9.35	164.13 ± 2.55	67.50 ± 1.37
Mean ± SD	236.80 ± 177.57	1349.34 ± 971.53	183.67 ± 135.98	162.31 ± 49.21	64.46 ± 57.74	43.88 ± 46.58
Range (min–max)	83.17–732.16	413.63–4026.40	65.80–567.27	55.83–233.00	21.83–199.33	4.70–173.77
Manuka	190.60 ± 0.85	1229.73 ± 1.03	809.73 ± 21.69	216.97 ± 10.27	45.23 ± 0.76	158.13 ± 1.72

Results are expressed as mean ± SD. n.d.: not detected.

**Table 5:** Trace elements in the different types of Malaysian honey samples.

Type of honey	As mg/kg	Pb mg/kg	Cd mg/kg	Cu mg/kg	Co mg/kg
Acacia	0.126 ± 0.030	1.017 ± 0.040	0.20 ± 0.00	2.11 ± 0.13	0.107 ± 0.005
Pineapple	0.062 ± 0.016	0.850 ± 0.042	0.80 ± 0.14	2.25 ± 0.14	0.101 ± 0.002
Gelam	0.064 ± 0.005	0.777 ± 0.012	0.05 ± 0.07	2.21 ± 0.02	0.082 ± 0.005
Longan	0.057 ± 0.003	0.723 ± 0.027	0.05 ± 0.07	2.42 ± 0.15	0.065 ± 0.001
Borneo	0.051 ± 0.001	0.588 ± 0.011	n.d.	2.83 ± 0.15	0.058 ± 0.003
Rubber tree	0.075 ± 0.002	0.029 ± 0.001	n.d.	2.61 ± 0.01	0.066 ± 0.001
Sourwood	0.028 ± 0.001	0.011 ± 0.002	n.d.	2.40 ± 0.52	0.069 ± 0.001
Tualang	0.062 ± 0.007	0.183 ± 0.007	n.d.	1.25 ± 0.63	0.033 ± 0.002
Trigona type 1	0.043 ± 0.002	n.d.	1.03 ± 0.25	n.d.	n.d.
Trigona type 2	0.027 ± 0.001	0.691 ± 0.002	0.78 ± 0.04	n.d.	n.d.
Rain forest	0.034 ± 0.002	n.d.	0.29 ± 0.01	2.46 ± 0.64	n.d.
Bitter gourd	0.041 ± 0.001	0.134 ± 0.014	0.88 ± 0.04	1.55 ± 0.69	0.023 ± 0.001
Trigona type 3	0.043 ± 0.001	n.d.	0.51 ± 0.01	2.40 ± 0.06	n.d.
Trigona type 4	0.052 ± 0.002	n.d.	0.33 ± 0.04	2.93 ± 0.36	n.d.
Mean ± SD	0.068 ± 0.025	0.36 ± 0.39	0.35 ± 0.38	1.96 ± 0.94	0.043 ± 0.028
Range (min–max)	0.027–0.126	n.d.–1.017	n.d.–1.03	n.d.–2.93	n.d.–0.107
Manuka	0.256 ± 0.002	n.d.	1.10 ± 0.71	2.17 ± 0.10	0.136 ± 0.007

Results are expressed as mean ± SD. n.d.: not detected.
